# Pedagogical Strategies for the Enhancement of Medical Education

**DOI:** 10.1007/s40670-021-01385-w

**Published:** 2021-08-27

**Authors:** Mohammad B. Azzam, Ronald A. Easteal

**Affiliations:** 1 Faculty of Education, Western University, London, ON N6G 1G7 Canada; 2 Department of Biomedical and Molecular Sciences, Queen’s University, Kingston, ON K7L 3N6 Canada

**Keywords:** Learning, Memory pathways, Schema, Pedagogical strategies

## Abstract

Clearly, memory and learning are essential to medical education. To make memory and learning more robust and long-term, educators should turn to the advances in neuroscience and cognitive science to direct their efforts. This paper describes the memory pathways and stages with emphasis leading to long-term memory storage. Particular stress is placed on this storage as a construct known as schema. Leading from this background, several pedagogical strategies are described: cognitive load, dual encoding, spiral syllabus, bridging and chunking, sleep consolidation, and retrieval practice.

## Introduction

“Learning represents the ability to use past experiences in the service of the present” [1]. The sum of past experiences is referred to as memory. Memory and learning are, together, essential to all aspects of human behavior and are particularly essential in all academic disciplines. Historically, medical students have regarded the discipline as one demanding rote learning as a key to success. Recent advances in learning and memory have led researchers to develop pedagogical strategies that allow both educators and learners to avoid rote memorization, as well as to build cognitive frameworks that promote life-long learning.

These cognitive frameworks include (1) the Circuit of Papez, by which new material is consolidated into long-term memory (LTM); (2) the formation of schemas, or the complex representations in the association cortex that both store memory and enable new material to be more efficiently and robustly absorbed; and (3) the retrieval of memory, or the reversal of the encoding and consolidation pathways of memory. Understanding these pathways would help clinicians in elucidating the location of potential disruption and aid in the diagnosis and treatment of the loss of cognition and memory. Furthermore, reviewing the neuroanatomy of memory and its pathways in this paper prior to describing the relevant pedagogical strategies would help readers/educators utilize these strategies more effectively and confidently convince their students of utilizing them.

Processes that are fundamental to the paradigms and pertinent to teaching and learning include (1) cognitive load; (2) dual encoding; (3) spiral syllabus; (4) bridging and chunking; (5) sleep consolidation; and (6) retrieval practice. This review describes the literature related to the neuroanatomy of memory, including its stages and pathways, and it also examines in detail the pedagogical strategies listed above.

## Neuroanatomy of Memory

Memory storage is located in multiple regions of the human brain. Most of the long-term memory (LTM) storage is in the parieto-temporal-occipital (PTO) junction of the association cortex, as well as the prefrontal cortex (PFC), on both its ventromedial and dorsolateral surfaces [2].

The Circuit of Papez, also known as the medial limbic circuit, is crucial to the consolidation of recent memory (RM) in the hippocampus (HPC) to LTM in the association cortex [3]. New memories undergo multiple passes through the circuit, thereby continually strengthening the LTM [3]. Simultaneously, the new stabilized memories become independent of the HPC. Restudying material enhances this process; however, its function in sleep consolidation is a much more effective promoter of LTM [4].

The cingulate gyrus is an essential component of the Circuit of Papez. It directs RM from the HPC into LTM in the association cortex. Repeated study (i.e., rereading and memorizing) of new content cycles the circuit multiple times, allowing for the connections of the cingulate gyrus to input memory to the LTM stores in the association cortex [3].

The small nucleus at the ventral tip of the HPC, the amygdala, plays a very important role in reward-related motivation. If a study session is seen to have a reward (i.e., better marks or feeling of achievement), it is the amygdala that is responsible [5].

## Memory Categories

Neuroanatomists categorize memory into four main types: (1) sensory memory (SM), or short-term memory (STM); (2) working memory (WM); (3) recent memory (RM; intermediate-term) and (4) LTM. LTM can be stored indefinitely within the cerebrum’s PTO junction dorsally, as well as within the PFC ventrally. There are two major categories of LTM [3]: explicit (declarative) memory and implicit (non-declarative; procedural) memory.

Implicit memory is defined as performing a familiar task automatically (i.e., riding a bike). Explicit memory, however, involves the recall of specific events or facts [3]. In recollection of an event, for example, a subset of explicit memory—episodic memory—is utilized. The memorization of facts, on the other hand, utilizes another subset of explicit memory—semantic memory. Use of semantic memory is by far the mode of choice for such disciplines as medical education [3]. This paper is devoted to semantic memory and the memory pathways involved in its encoding, consolidation, and retrieval.

**Fig. 1:**
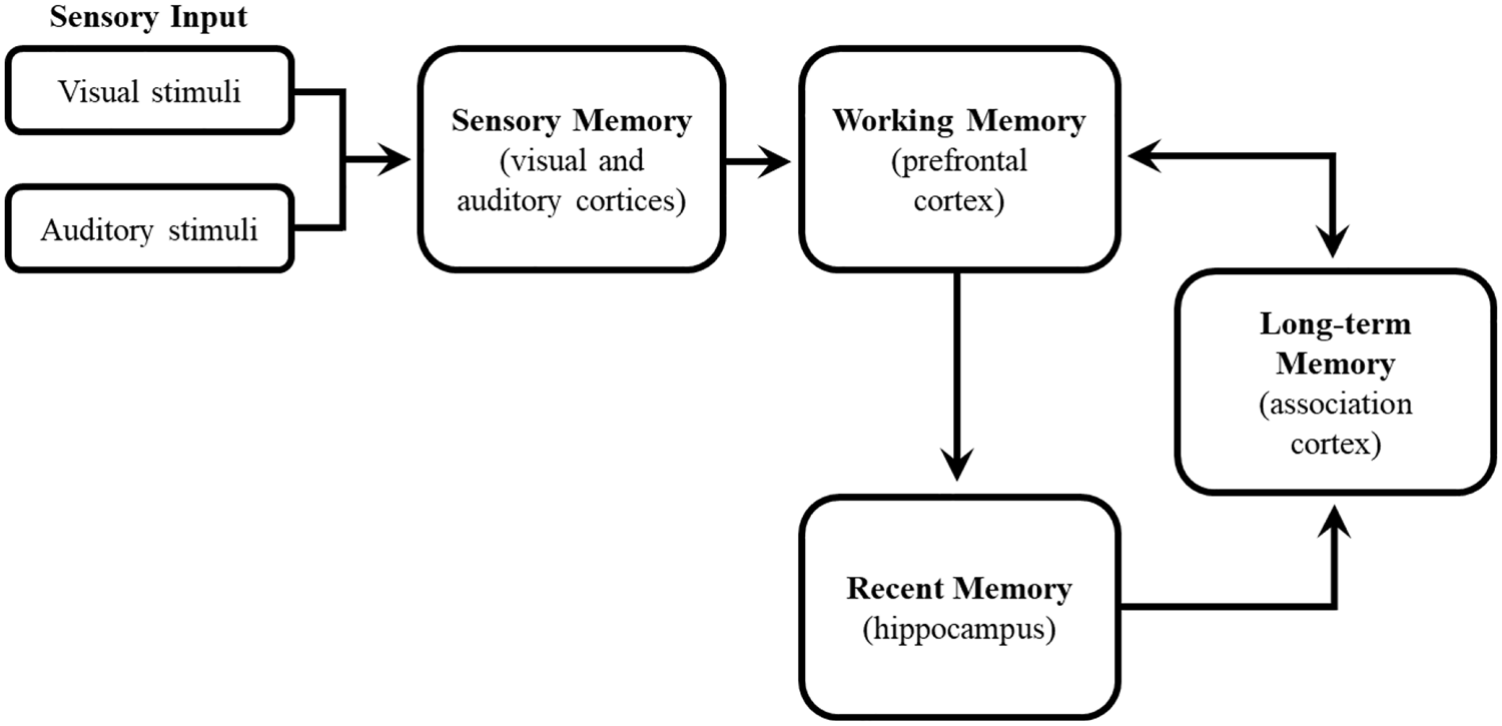
The progression of new memory into permanent LTM storage in the association cortex

The progression of new memory into permanent LTM storage in the association cortex is shown in Fig. 1. It begins with SM, which lasts for a few seconds, to WM, which lasts for approximately 12 seconds. WM is then transferred to RM within the HPC, where it resides for up to a week, and then into the more permanent LTM [3]. The pathways involved are encoding, consolidation, and retrieval. Encoding transfers WM into both RM and LTM simultaneously. Subsequent consolidation transfers RM in the HPC to LTM. Of course, for any of this to be useful, the memory must be successfully retrieved back into WM.

### Sensory Memory

Most knowledge, which is eventually stored as semantic LTM, is acquired by the human brain via images and speech [6]. These two sensory modalities enter the visual and auditory cortices as SM, which is only available for a few seconds and is then transferred into WM [7] (Fig. 1). Each of these two modalities has a very limited capacity both in terms of content and of time. These limitations have an important bearing on teaching and learning, and will be addressed later.

### Working Memory

WM takes the input from SM, manipulates it, and then encodes it into both RM (in the HPC) and LTM (in the association cortex) simultaneously. It gives us the ability to hold and manipulate new information, allowing for its integration with retrieved, congruent content in LTM [8]. The input from SM is mostly in the form of visual and auditory information (Fig. 1). This incoming content is processed and then analyzed by a component of WM—the central executive, which is located in the dorsolateral prefrontal cortex (DL-PFC). The central executive analyzes this input leading to encoding into both the HPC as RM and into the association cortex as LTM [9, 10]. WM is limited, both in terms of content and longevity. It can store only 5–7 items at a time, for no longer than 12 seconds [11]. If the new information is congruent with schemas from LTM, then the central executive retrieves this congruent content from the LTM (Fig. 1), combines the new information with the old, and re-encodes them together, creating more robust memory [9, 10]. Moreover, the fact that WM has a limited capacity will reflect upon the concept and utilization of cognitive load applications, which will be discussed later.

### Recent Memory

WM products are encoded into the HPC in the form of engrams. This memory is classed as RM (Fig. 1). It is then stored in the HPC for a limited time (a few days) [12]. In a seminal paper, Scoville and Milner [13] described the consequences of bilateral removal of the HPC of Henry Molaison (H.M.) to alleviate intractable seizures. The surgery accomplished the intended aims, but left H.M. with the inability to form new memories; he also lost the memories he acquired a few days before the surgery. Scoville and Milner correctly assessed that the HPC was essential for acquiring new memory and that it stored the memory for a few days. RM is gradually consolidated into LTM for permanent storage (Fig. 1). It is aided in this process by interacting with the ventromedial prefrontal cortex (VM-PFC) [14].

### Long-term Memory

LTM is gradually consolidated from RM over a few days (Fig. 1). It is stored in specialized representations within the PTO junction known as schemas [3]. These regions correspond to the association loci of the areas that first received the visual and auditory input in the PTO cortex as well as the VM-PFC. LTM may be stored for many years but may weaken over time; if, however, consolidation and reconsolidation are practiced, the memory will become more robust and can be more easily retrieved [3]. Fundamental to the encoding, consolidation, and retrieval of information are the constructs of engrams and schemas. These constructs rationalize the packaging, storage, and manipulation of learning.

## Engrams and Schemas

It is generally agreed by neuroscientists that engrams are formed as strengthened connections between neuron assemblies during the process of encoding [15]. Engrams consist of strongly connected cortical cell bodies that can be integrated into existing long-term memory schemas. As far back as 1933, Bartlett posited that memory and understanding were contained within the cortex as a framework of interlinked mental structures [16]. In 1952, Piaget introduced the term “schema” to describe such frameworks [17]. Each schema is an associative network comprised of a very complex group of interconnected engrams that are activated during mnemonic processing. Formation of engrams and schema forms the basis of learning and the understanding of these structures and pathways will substantially potentiate learning efficiency. Schemas are extremely important in teaching and learning. If new information is related to an existing schema, then that new, congruent information is more easily encoded and is, therefore, more easily memorized. Setting up individual bits of information into their own schemas aids mnemonic processing. This teaching strategy is known as chunking and is particularly useful in medical education.

## Memory Pathways

### Encoding

**Fig. 2:**
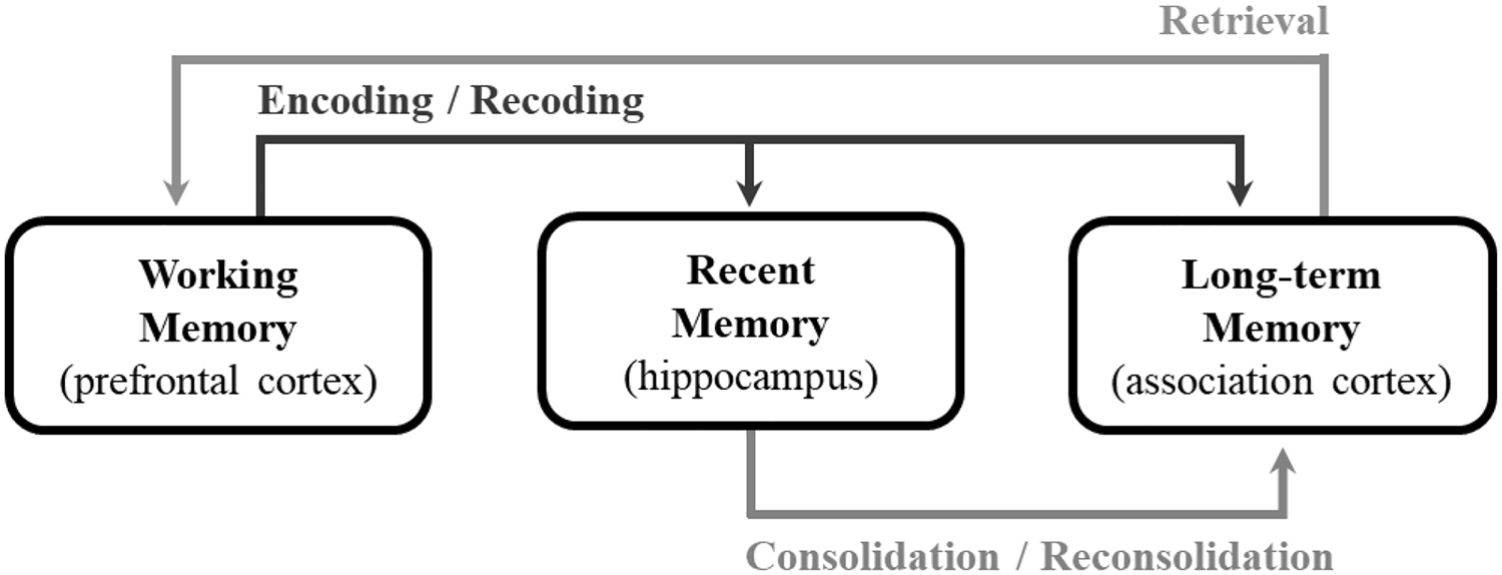
The three memory pathways: encoding, consolidation, and retrieval

Encoding connects WM to RM in the HPC. It also weakly connects to LTM within the association cortex as engrams (Fig. 2). Encoding of the WM engrams directly to LTM creates rather weak schemas of the recently added information within the association cortex. These schemas are specific for recently added information [18]. Because the same information is encoded to RM simultaneously, hippocampal engrams of that information are created, as well [19]. Next, the RM engram is transferred from the HPC to the parts of the association cortex that serve LTM—a process known as consolidation (Fig. 2).

### Consolidation

There are two classifications of consolidation: (1) synaptic consolidation and (2) systems consolidation. Synaptic consolidation is the strengthening of neural networks between the hippocampal (presynaptic) neurons and the cortical (postsynaptic) neurons. Synaptic consolidation occurs via a process known as long-term potentiation (LTP). When synapses are repeatedly activated (presumably, by repeated restudy), LTP leads to the generation of a protein cascade in the cortical neurons and, consequently, increases the strength of the connection between the hippocampal and cortical neurons. This process, first elucidated by Donald Hebb [4], has been described as “neurons that fire together, wire together.” Furthermore, LTP is seen as a major factor in sleep consolidation, which is described later. Systems consolidation, on the other hand, describes the integration of RM from the HPC into the long-term stores of the association cortex. Clearly, repeated study engages the Circuit of Papez multiple times; thus, the connections between the cingulate gyrus and the association cortex undergo LTP, which leads to synaptic memory consolidation.

Standard Systems Consolidation (SSC), on the other hand, describes the interconnectivity of the hippocampal schemas (or RM) and the cortical schemas (or LTM) of a memory, which results from previous encoding (Fig. 2). Over several days, the HPC guides a process that further binds the cortical modules into a schema. Squire et al. [20] posit that as memories mature, the cortical schemas recruit a “hub” in the association cortex—the VM-PFC. This recruitment of the VM-PFC has been found to be critical in relating new memory to existing schemas and in the retrieval of memory [21, 22]. Eventually, the HPC becomes disengaged and the hippocampal-cortical connections are eventually dropped [20]. This process strengthens the memory’s weak representations within the association cortex, making it more robust.

Furthermore, memories that have been systematically consolidated with well-established schemas are much easier to recall than pre-consolidated (i.e., merely encoded) memories [22]. This phenomenon is known as the schema effect. Encoded memory is, however, more easily recalled if the new input is associated with previously existing schemas. This has a bearing on teaching and learning in that if new information is “bridged” to previously learned content, it is more easily absorbed and remembered, even after a short encoding period.

### Retrieval

In order for LTM to be of any use, it must be retrieved. The retrieval of semantic memory is thought to be the reversal of the encoding and consolidation pathways from LTM to WM [23] (Fig. 2). If retrieval is required for a semantic memory, the memory is simply retrieved into WM and is used to answer the question at hand [24]. Furthermore, retrieval is extremely important, especially when evaluating new information that is congruent with existing schemas. A germane (congruent) schema is retrieved into the central executive of WM and analyzed in conjunction with the new input, as described previously. Consolidation and retrieval of congruent information form the cognitive base of several pedagogical strategies, as discussed next.

## Teaching Strategies

The strategies presented below are techniques based on the structure and function of the neurocognitive pathways presented above. They provide optimal utilization of connecting new information into human memory pathways to obtain the best possible outcomes. The main techniques include (1) cognitive load; (2) dual encoding; (3) spiral syllabus; and (4) bridging and chunking.

### Cognitive Load

Cognitive Load Theory (CLT) is the “brain child” of John Sweller [25]. Cognitive load refers to the degree of difficulty encountered by a learner in acquiring new knowledge. It reflects the fact that the input into WM is extremely limited in both capacity (5–7 items) and duration (12 seconds) [11]. Instructors should, therefore, accommodate for these limitations. According to the CLT, cognitive load is comprised of three components: intrinsic load, germane load, and extraneous load.

Intrinsic load refers to the information that is essential to the learner. For example, in teaching the location of the aorta as it passes through the diaphragm, the intrinsic load comprises only the information that it does so at T12 in an opening between the left and right diaphragmatic crura.

Germane load is related to the schema construction, whereby the new intrinsic load is integrated into LTM, either by creating a new schema or by associating new information with existing schemas. This may be represented as the mode of presentation of new information. In the example above, germane load is embodied in the presentation of visual and verbal information simultaneously (refer to Fig. 1); a diagram of the aorta as it passes through the diaphragm should be accompanied by a verbal description.

Extraneous load is defined by the mental effort wasted by the use of elements that do not contribute to the formation of relevant schema. In the above example, presenting information such as the neurovasculature of the diaphragm would constitute extraneous load. Input of intrinsic and germane loads, while eliminating the extraneous load, would prove beneficial to learning of congruent information without inflicting unnecessary work on memory formation.

### Dual Encoding

Dual encoding is the optimization of the two sensory modalities (visual and auditory) into SM and then into WM (Fig. 1) [26]. Given that the capacity of WM is limited for both visual and auditory learning, each of these modalities is, in turn, limited. Therefore, if a diagram is shown with too many printed labels (i.e., during a lecture), the visual input is compromised. If, however, the diagram is shown with spoken description, thereby having the students label the diagram themselves, the WM can manage both inputs simultaneously. This method comes hand-in-hand with CLT; labelling structures unnecessarily imposes high extraneous load.

### Spiral Syllabus

Jerome Bruner [27] posited that if new learning were constructed as a well-established base, that new information would be better learned. The spiral syllabus is one in which learning occurs in a logical, sequential manner, going from a simple base to expand to a comprehensive understanding of the intended learning outcomes of the course or program.

### Bridging and Chunking

Both bridging and chunking are teaching modalities that are based on schema-based learning. Bridging is the process by which germane material is reintroduced as a building block for the ensuing class. In so doing, students retrieve relevant semantic memory and link the old memories with the new information. Thus, when students retrieve one of these memories during formal examination, for example, they would be able to retrieve all these memories together. Furthermore, every syllabus and, indeed, every class, can be broken down into a series of chunks, which allows for easier digestion of learned material and easier ability to connect related ideas together. Both bridging and chunking are perfectly in line with the teaching and learning of medical disciplines such as anatomy. For example, the anatomy of the arm and forearm are typically taught in subsequent lectures. This arrangement allows for bridging, as both regions of the upper limb have common neurovasculature (e.g., radial nerve). Hence, reviewing the radial nerve’s innervation of the triceps brachii muscle before discussing its innervation of the posterior compartment of the forearm is imperative to effective retrieval (Fig. 2) of all semantic memory regarding the radial nerve. Furthermore, the lecture on the forearm can be divided into two major chunks: (1) the anterior compartment of the forearm and (2) the posterior compartment of the forearm, whereby each compartment’s dedicated muscle actions and neurovasculature are discussed.

## Learning Strategies

Educators and students utilize learning strategies to take advantage of human memory and memory pathways to improve life-long learning. Two of the most important learning strategies are (1) post-encoding sleep consolidation and (2) retrieval practice.

### Post-encoding Sleep Consolidation

Sleep consolidation is Mother Nature’s gift for learning. It is a process whereby the connections between RM in the HPC and the LTM in the association cortex (Fig. 1) are made much more robust. The consequence of this process is that semantic memory is better-retrieved in the morning (after sleep), compared to when the material was learned (before sleep). Feld and Diekelmann [28] state that “long-term memory is formed during sleep by a process that strengthens memory traces, reorganizes them, and integrates them into established knowledge networks.”

Sleep is divided up into two basic stages: rapid eye movement (REM) sleep and non-REM sleep. Non-REM sleep itself has four stages; the fourth and deepest stage of non-REM sleep produces cortical slow waves and is therefore labelled as slow-wave sleep (SWS). In an 80-min sleep cycle, approximately 30 min are devoted to SWS alone. It is during SWS that the transfer of information from the HPC to the association cortex is activated. During sleep, the brain is considered to be “offline” when there is no sensory input to the major sensory nucleus—the thalamus. The thalamus at this time, however, is not quiescent; rather, it produces a series of waves—the thalamic spindles. Simultaneously, the HPC produces sharp-wave ripples—K-complexes [29]. Staresina et al. [30] demonstrate that the thalamic spindles cluster the hippocampal K-complexes for precisely timed incorporation of information into the association cortex’s slow waves.

These incorporated spindles during the up states of slow waves are thought to initiate rapid repetition of LTP in the association cortex. According to Klinzing et al. [31], “Repeated neuronal replay of [schemas] originated in the HPC during slow-wave sleep leads to a gradual transformation and integrations of [schemas] in neocortical networks.” This repeated process of synaptic consolidation eventually leads to systems consolidation and the resulting enhancement of LTM in the neocortex with concomitant loss of hippocampal RM (Fig. 2). A typical sleep cycle lasts for 80 min, 30 min of which is SWS. Each slow wave is about 1 s long and receives 5–7 ripples in its up state. Thus, it is possible that in 30 min of deep sleep, LTP could be triggered 9000 times (5 ripples × 60 s × 30 min)! This rapid repetition of synaptic consolidation gives rise to robust and active systems consolidation.

It is important to realize that post-encoding sleep consolidation not only consolidates factual (semantic) information, but also extracts the important concepts of previously encoded schemas, thereby enabling the learner to synthesize new information with previously learned factual or analytical schemas.

Another feature of sleep consolidation is that it is selective; Wilhelm et al. [32] showed that sleep preferentially encodes material that is relevant to the student’s learning. For example, material that has been emphasized by the educator as important is more likely to be encoded. Information is also more likely to be recorded during sleep consolidation if a perceived reward exists; the reward can either be intrinsic (i.e., in terms of student satisfaction) or extrinsic (i.e., in terms of improved grades).

### Retrieval Practice

Retrieval practice (RP), as a learning tool, has been studied for over a hundred years [33]. Modern day interest in RP has been propagated out of Washington University in St. Louis, MO, by Jeffery Karpicke and Henry Roediger III [34]. RP can be defined as the reactivation of neural pathways by students attempting to recall (retrieve) previously encoded information from LTM schemas into WM. The use of RP has been found to be of greater mnemonic value than simply repeated study of coursework material [35–39]. The mnemonic benefit has been labelled as the testing effect, and has been studied in the laboratory setting with word-pair testing as well as in the classroom.

There are two overlapping theories that pertain to the mechanisms of RP’s effectiveness: (1) the Standard Consolidation Model (SCM) and (2) the Multiple Trace Theory (MTT). The SCM states that memories become independent of the HPC post-consolidation [40], while the MTT argues that the HPC is necessary for retrieval (Fig. 2), particularly of episodic memories [41].

Furthermore, Wing et al. [42] found that the testing effect may be contingent on processes that support the memory’s success during encoding. This is supported by van Kesteren et al. [14] who demonstrated that neural networks that are active in encoding and consolidation are reactivated during retrieval. These studies provide, at the very least, a theoretical rationale for the efficacy of RP.

RP has been heavily researched in a controlled setting. Under such circumstances, RP has been proven to be a superior mnemonic device [1]. The question to be answered is “How does RP work in a classroom setting over an entire academic semester?” Although there is less research that assesses the effectiveness of RP in the classroom, the evidence that does exist suggests that this learning tool can be successfully transferred to the classroom [43].

When compared to repeated study of coursework material, RP constantly improves long-term retention [44]. Moreover, the scheduling of RP has been shown to be of critical importance. RP is best done on an expanded schedule (i.e., 2 days, 1 week, 3 weeks). Not only does expanded retrieval correlate with improved grades, but it also allows more material to be memorized. This is a very important consideration in medical disciplines, where factual information is delivered on a continuous basis.

In addition, RP questions should make the students think about the answers, thereby requiring effortful recall. Therefore, RP questions should be asked in the form of production tests (i.e., short answer questions), rather than recognition tests (i.e., multiple-choice questions).

Furthermore, RP should be implemented at the beginning of the next lecture if the next lecture contains material that can be “bridged” back to the previous lecture. Memory recalled from LTM in this method returns to WM, and is therefore subject to alteration (i.e., addition). In other words, the memory is in a labile condition. As stated before, if congruent material is added to this juncture, the eventual LTM will be enhanced [14].

Another consideration for RP is that of feedback. It is critical for RP that the correct answers be provided; the question to be answered is “When?” In the case above, where RP relates directly to the congruent material that is being taught, feedback should be given before new, congruent material related is presented. In other cases, where information is not related, feedback should be delayed [45].

The main challenge faced by educators when implementing RP is convincing the students of RP’s effectiveness. Students have been used to repeated study of coursework material for their entire lives; therefore, they are usually distrustful of any other learning strategies, perhaps in part due to their lack of metacognitive awareness of the testing effect [46]. It behooves the educator to overcome this challenge and convince the student population that RP does, indeed, work.

## Conclusion

This paper has drawn together the pedagogical strategies that are based upon the latest research into neuroanatomy and cognitive science. Memory stages and pathways were described, along with their anatomical substrates. Particularly important is the nexus between new information and LTM and the way that the schemas in LTM integrate the new information. This creates a far more robust scaffold.

The limitations of SM and WM, particularly, restrict the input of new information. This should be understood by instructors in the teaching of new information. Rote memorization does not promote life-long learning. For those instructors, particularly in disciplines where rote memorization has been the accepted standard, turning to new teaching and learning strategies that promote long-term memory retention can improve student experience and contribute to their advancement going forward.

## Abbreviations

CLT: Cognitive Load Theory
DL-PFC: Dorsolateral prefrontal cortex
H.M.: Henry Molaison
HPC: Hippocampus
LTM: Long-term memory
LTP: Long-term potentiation
MTT: Multiple Trace Theory
PFC: Prefrontal cortex
PTO: Parieto-temporal-occipital
REM: Rapid eye movement
RM: Recent memory
RP: Retrieval practice
SCM: Standard Consolidation Model
SM: Sensory memory
SSC: Standard Systems Consolidation
STM: Short-term memory
SWS: Slow-wave sleep
VM-PFC: Ventromedial prefrontal cortex
WM: Working memory
